# Research progress of heat stroke during 1989–2019: a bibliometric analysis

**DOI:** 10.1186/s40779-021-00300-z

**Published:** 2021-01-21

**Authors:** De-Meng Xia, Xu-Ren Wang, Pan-Yu Zhou, Tian-Le Ou, Lei Su, Shuo-Gui Xu

**Affiliations:** 1 Department of Emergency, Changhai Hospital, the Naval Medical University, Shanghai, 200433 China; 2 Nursing College, The Naval Medical University, Shanghai, 200433 China; 3 Department of Clinical Medicine, The Naval Medical University, Shanghai, 200433 China; 4 Department of Intensive Care Unit, General Hospital of Southern Theater Command, Guangzhou, 510010 China

**Keywords:** Heat stroke, Publications, Citation frequency

## Abstract

**Background:**

Heat stroke (HS) is an acute physical disorder that is associated with a high risk of organ dysfunction and even death. HS patients are usually treated symptomatically and conservatively; however, there remains a lack of specific and effective drugs in clinical practice. An analysis of publication contributions from institutions, journals and authors in different countries/regions was used to study research progress and trends regarding HS.

**Methods:**

We extracted all relevant publications on HS between 1989 and 2019 from Web of Science. Using the Statistical Package for Social Science (SPSS, version 24) and the software GraphPad Prism 8, graphs were generated and statistical analyses were performed, while VOSviewer software was employed to visualize the research trends in HS from the perspectives of co-occurring keywords.

**Results:**

As of April 14, 2020, we identified 1443 publications with a citation frequency of 5216. The United States accounted for the largest number of publications (36.2%) and the highest number of citations (14,410), as well as the highest H-index at 74. Although the sum of publications from China ranked second, there was a contradiction between the quantity and quality of publications. Furthermore, *Medicine & Science in Sports & Exercise* published the most papers related to HS, with Lin MT publishing the most papers in this field (112), while the review by Knochel JP received the highest citation frequency at 969. The keyword heat-stress appeared most recently, with an average appearing year of 2015.5. In the clinical research cluster, exertional heat-stroke was determined to be the hotspot, while ambient-temperature and heat waves were the new trends in the epidemiological research cluster.

**Conclusions:**

Corresponding to this important field, while the contributions of the publications from the United States were significant, the mismatch between the quantity and quality of publications from China must be examined. Moreover, it is hypothesized that clinical and epidemiological studies may become hotspots in the near future.

**Supplementary Information:**

The online version contains supplementary material available at 10.1186/s40779-021-00300-z.

## Background

Heat stroke (HS), considered one of the most fatal diseases, is commonly depicted by an unbalanced generation and dissipation of heat as a result of exposure to a hot environment or strenuous exercise, and itis clinically characterized by central nervous system (CNS) dysfunction, multiple organ failure and extreme hyperthermia (usually> 40.5 °C) [1]. Despite decades of research, it is still clinically recommended to appropriately lower the body temperature and engage in active treatment measures, even though HS is often fatal, with the mortality rate in the elderly exceeding 50% [2]. At present, the main studies on HS focus on the pathological changes of decompensation caused by the body under thermal stimulation and on the related mechanisms of systemic inflammation and multiple organ failure caused by HS [3]. Unfortunately, as a result of the consistent deterioration caused by global warming, the number of people dying from heat waves continues to grow. For example, during August 2003, a sustained severe heat wave in Europe resulted in 14,800 heat-related deaths in France [4]. According to another study, the relative risk (RR) of HS, which is related to the length and density of the heat wave, on heat wave days compared to that on matched nonheat wave days decreased from 71.0 in 1999 to 3.5 in 2010, further suggesting a close relationship between the incidence of HS and ambient temperature [5]. Consequently, research regarding HS is vital for correlative countries and regions.

Bibliometrics is a statistical and quantitative method used to analyze the academic influence and characteristics of scientific output. Combined with creative design and information visualization, bibliometric mapping visually represents bibliometric data [6], highlights the impacts of a given study on a discipline, and potentially promotes an understanding of the data. In addition, bibliometrics has been widely used in the fields of information science, chemistry and physics, with new potential prevailing in the field of medicine [7]. Using bibliometrics, researchers can determine more specific research themes and thereby achieve a more comprehensive understanding of the relationships between specific research areas.

The present study was aimed at comprehensively analyzing the research progress with respect to HS based on Web of Science (WOS). We applied a bibliometric analysis for the purpose of uncovering the research trends related to HS and predicting its possible future hotspots. Although Mao et al. [8] used bibliometric methods to report the publication trends of exertional HS from 1996 to 2015, a comprehensive review of the publication trends and future hotspots covering HS still require further exploration.

## Materials and methods

### Data sources and search strategies

It has been unanimously accepted that the Science Citation Index-Expanded (SCI-E) of Thomson Reuters’ WOS is one of the most appropriate databases for conducting bibliometric analysis research. Therefore, a comprehensive online search dated from 1989to2019 was conducted in WOS with publication types limited to original articles and reviews. As all data were obtained from the public database and had nothing to do with any human subject, ethical consent was not applicable.

**Fig. 1:**
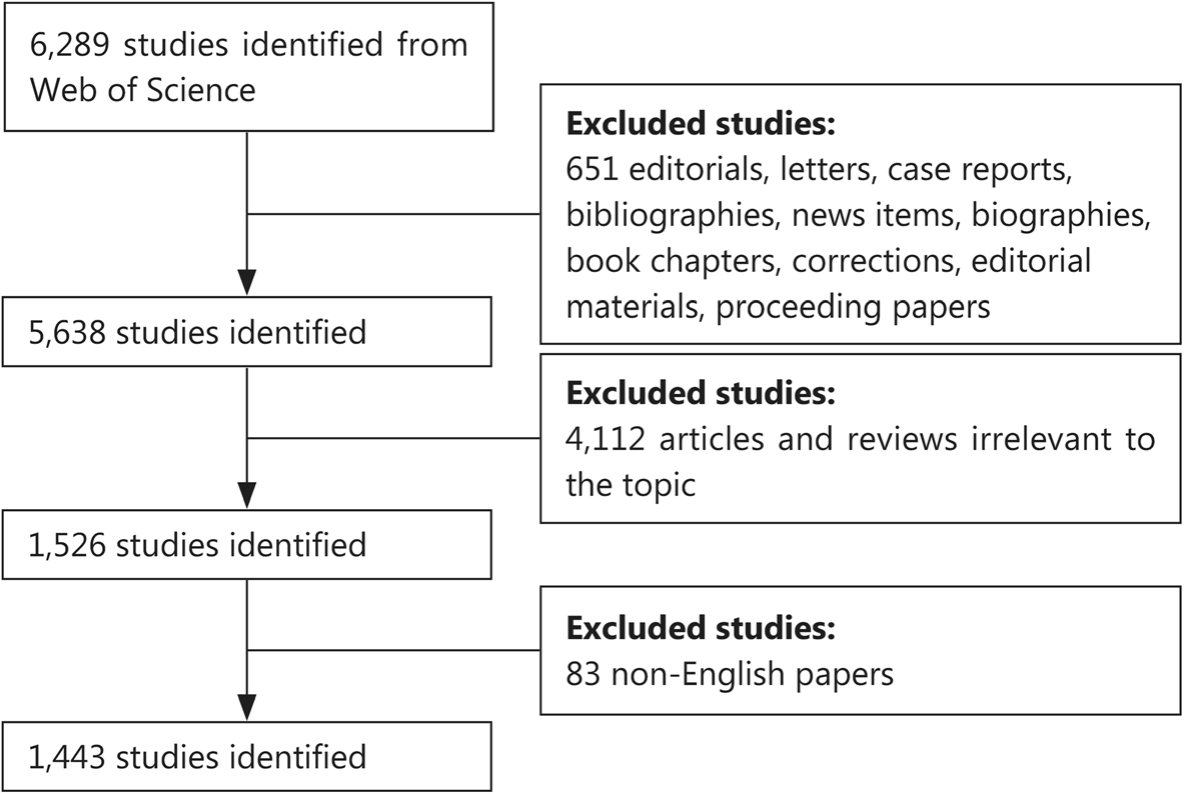
Flow diagram of the inclusion process. The detailed process of screening and enrollment

All searches were conducted on a single day, April 14, 2020, to avoid omissions arising from rapid database renewal. The search strategies were presented as follows: TS = ((heat stroke) OR (stroke heat) OR (heat strokes) OR (heat apoplexy) OR (heatstroke) OR (thermoplegia) OR (heat hyperpyrexia) OR (heatstrokes) OR (heat-stroke) OR (non-pyrogenic hyperthermia) OR (heat illness)) AND Language = English. Original articles and reviews with normal peer-review were potentially eligible, while others were excluded accordingly. Detailed processes of enrollment and screening are displayed in Fig. 1.

### Data collection

Two authors (TLO and DMX) independently extracted data from all correlative publications, including titles, keywords, publication dates, origin countries and regions, authors, institutions, published journals, sum of citations, H-index, and so on. The data from WOS was inputted into the statistical package for social sciences (SPSS, version 24, IBM Corporation, USA), Microsoft Excel 2016 (Redmond, Washington, USA), GraphPad Prism 8 (GraphPad Prism Software Inc., San Diego, CA), and VOSviewer version 1.6.12 (Leiden University, Leiden, the Netherlands) and were subsequently analyzed and presented both quantitatively and qualitatively. Meanwhile, the World Bank website [9] and the Central Intelligence Agency (CIA) website [10] were retrieved for the latest information regarding the gross domestic product (GDP) and population.

### Bibliometric analysis

We applied WOS to analyze the characteristics of all incorporated publications. The term relative research interest (RRI) was defined as the number of publications concerning a specific research field divided by publications across all fields per year, while the impact factor (IF) was obtained from information published in the latest version of Journal Citation Reports (JCRs). It has been widely accepted that the H-index functions as a reflection of the scientific research impacts of a scholar or a country and that it indicates that a scholar or country has published H papers and that each of them has been cited in other publications at least H times.

VOSviewer is an optimal approach to analyzing the correlation of highly cited publications with productive authors. The viewer can be employed to construct maps of authors and journals based on cocitation data as well as maps of keywords based on cooccurrence data related to HS. Furthermore, VOSviewer can classify keywords into different clusters in accordance with the results of cooccurrence analysis and simultaneously color them by time course. The definition of average appearing year (AAY) was employed to quantify the relative novelty of a keyword. Hence, author cocitation analysis (ACA) was presented as a network visualization map by using VOSviewer (version 1.6.12) techniques [7], and the division of different clusters was defined as keywords in related research fields, and their frequencies of occurrence were calculated.

## Results

### Contributions of countries/regions to global publications

**Fig. 2:**
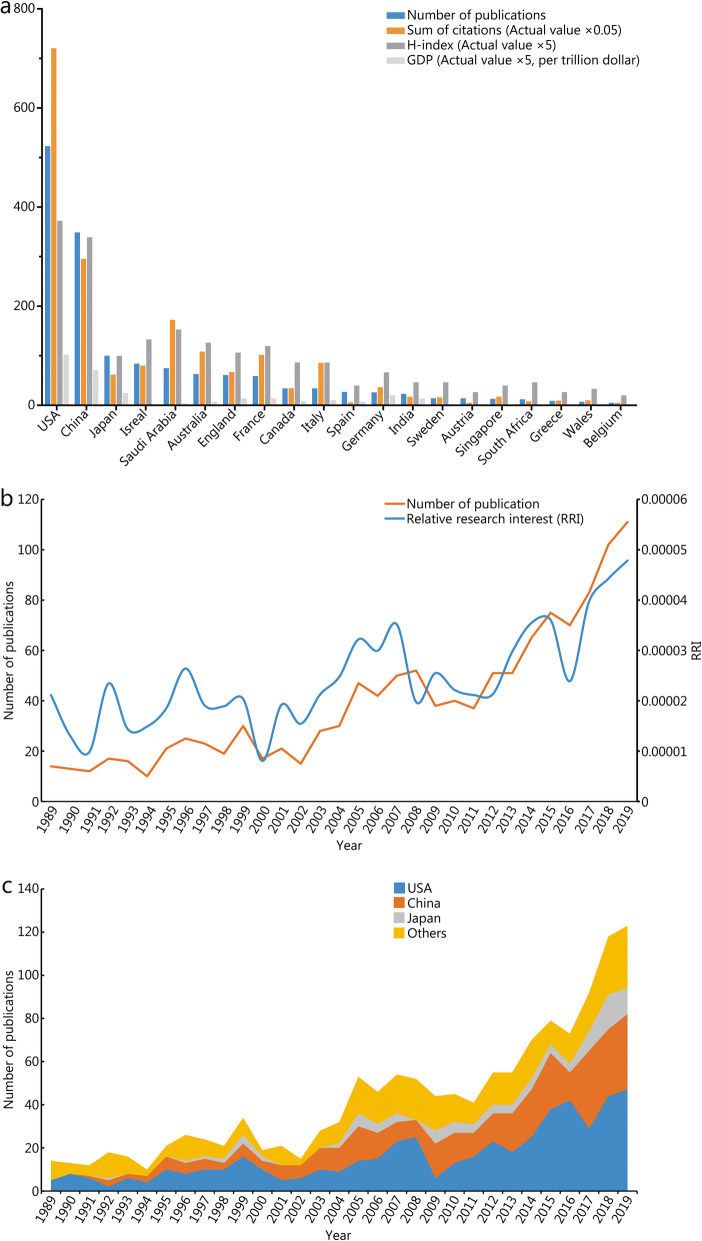
Contributions of different countries/regions to the research field regarding HS. **a** The number of publications, citation frequency (×0.05), H-index (×5) and GDP (×5, per trillion dollar) in the top 20 countries or regions; **b** The number of publications worldwide and the time course of relative research interest of HS; **c** The number of publications from the top three and other countries per year. RRI. Relative research interest

In all, 1443 articles dated from 1989 to 2019 met our inclusion criteria, with the United States ranking first in the number of publications at 523 (36.2%), followed by China at 349 (24.2%) and Japan at 100 (6.9%, Fig. 2a). By comparing the number of papers published per year, we found that the largest number of publications occurred in 2019, with 111 publications (7.8%, Fig. 2b). When the numbers of all-field publications were considered, the global interest in HS measured by the value of RRI fluctuated approximately 0.003% before 2014, but subsequently increased to 0.008% in 2019 (Fig. 2b). Not until 2001 did Chinese researchers gradually publish papers in this field. However, the proportion of Chinese publications in this field rose rapidly for the past 10 years (Fig. 2c).

### Citations and H-index analysis

By retrieving the Journal Citation Report from the WOS database, all articles related to HS had been cited 29,160 times since 1989 (22,166 times without self-citations), with an average citation frequency of 23.78 times per paper. The United States accounted for 49.3% of the total citations, i.e., 14,400 times (11,902 times without self-citations), and exhibited an H-index of 74. The number of citations from China was 7933 (4677 times without self-citations) with an H-index of 74, and thus ranked second among all involved countries and districts. **(**Fig. 2a**)**.

### Journals with research publications on HS

**Fig. 3:**
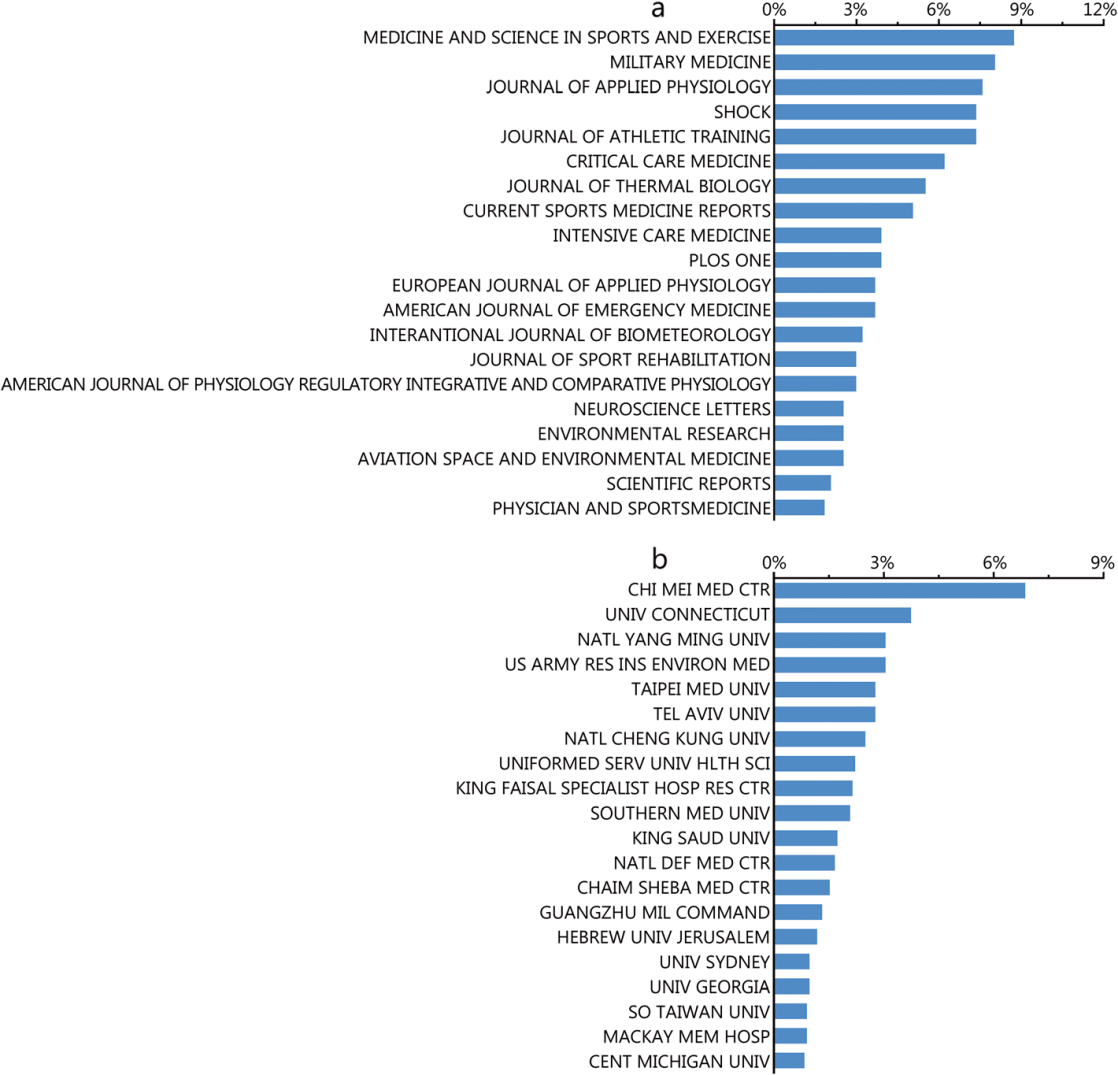
Distribution of institutions and journals focused on HS. **a** Distribution of top 20 journals publishing research on HS; **b** Distribution of top 20 institutes publishing research on HS

Approximately one-third of the papers within the relevant scope were published in the top 20 journals based on IF (399, 27.65%). Specifically, the number of papers published in *Medicine & Science in Sports & Exercise* (IF = 4.478, 2018) was the highest with 38 records, while the *Journal of Applied Physiology* (IF = 3.140, 2018) ranked third with 33 publications. Additionally, *Critical Care Medicine* (IF = 6.971, 2018) reported27 publications and was ranked sixth, and the journal of *Intensive Care Medicine* (IF = 18.967, 2018) ranked nineteenth, with a total of 17 publications. For other journals with immense academic impact, *Nature Biotechnology* published a high-quality article in a related field [11], and the *New England Journal of Medicine (NEJM)* published a review [3]. We also found a review that was published in the journal *Cell* [12]*.* The top 20 journals with the most publications are listed in Fig. 3a.

### Institutions with research publications on HS

The Chi Mei Medical Center in Taiwan Province, China had the highest number of publications among institutions worldwide, with 99 papers documented by this affiliation, which accounted for 6.7% of all publications. Within the list of top 20 institutions in this field, nine were Chinese institutions, five were institutions in the United States, three were Israeli institutions, two were Saudi Arabian institutions and one was an Australian institution (Fig. 3b).

### Authors with research publications on HS

A total of 381 papers written by the top ten authors accounted for 26.4% of all studies in the related area. Lin MT from the Chi Mei Medical Center published 112 papers related to HS, thus ranking first in the number of publications. Ranking second was Casa DJ, who had 46 papers. As presented in Table 1, there are five authors from China, three from the United States, one from Israel, and one from Saudi Arabia. Notably, Lin MT from the Chi Mei Medical Center had the highest citation frequency (2538, Tables 1, 2).

**Table 1:** Top 10 authors with most publications in research scope of HS

Corresponding author	Country	Affiliation	Publications (*n*)	Citations (*n*)
Lin MT	China	Chi Mei Medical Center	112	2538
Casa DJ	USA	University of Connecticut	46	1897
Su L	China	Southern Medical University	40	385
Leon LR	USA	US Army Research Institute of Environmental Medicine	36	1032
Chang CP	China	Chi Mei Medical Center	32	579
Armstrong LE	USA	University of Connecticut	28	1896
Bouchama A	Saudi Arabia	King Faisal Specialist Hospital	27	2284
Epstein Y	Israel	Tel Aviv University	24	741
Chen SH	China	Chi Mei Medical Center	22	403
Chang CK	China	Taipei Medical Hospital	14	332

**Table 2:** Top 10 high cited papers related to HS

Title	Corresponding author	Journal	Publication year	Total citations(*n*)	Average citations per year(*n*)	Corresponding author‘s country
Heat stroke	Knochel JP	*New England Journal of Medicine*	2002	969	57	USA
Stress-inducible responses and heat shock proteins: New pharmacologic targets for cytoprotection	Morimoto RI	*Nature Biotechnology*	1998	460	22	USA
Exertional heat illness during training and competition	Armstrong LE	*Medicine and Science in Sports and Exercise*	2007	443	37	USA
Human circulatory and thermoregulatory adaptations with heat acclimation and exercise in a hot, dry environment	Nielsen B	*Journal of Physiology-London*	1993	435	17	USA
Excess mortality related to the August 2003 heat wave in France	Hémon D	*International Archives of Occupational and Environmental Health*	2006	385	30	France
Excess hospital admissions during the July 1995 heat wave in Chicago	Semenza JC	*American Journal of Preventive Medicine*	1999	335	17	Sweden
The 2006 California Heat Wave: Impacts on Hospitalizations and Emergency Department Visits	Knowlton K	*Environmental Health Perspectives*	2009	321	32	USA
Heat-related mortality during a 1999 heat wave in Chicago	Naughton MP	*American Journal of Preventive Medicine*	2002	236	14	USA
The 2003 heat wave in France: Dangerous climate change here and now	Poumadère M	*Risk Analysis*	2005	235	17	France
National Athletic Trainers’ Association position statement: Exertional heat illnesses	Binkley HM	*Journal of Athletic Trainning*	2002	230	14	USA

### Analysis of keywords in publications of HS

**Fig. 4:**
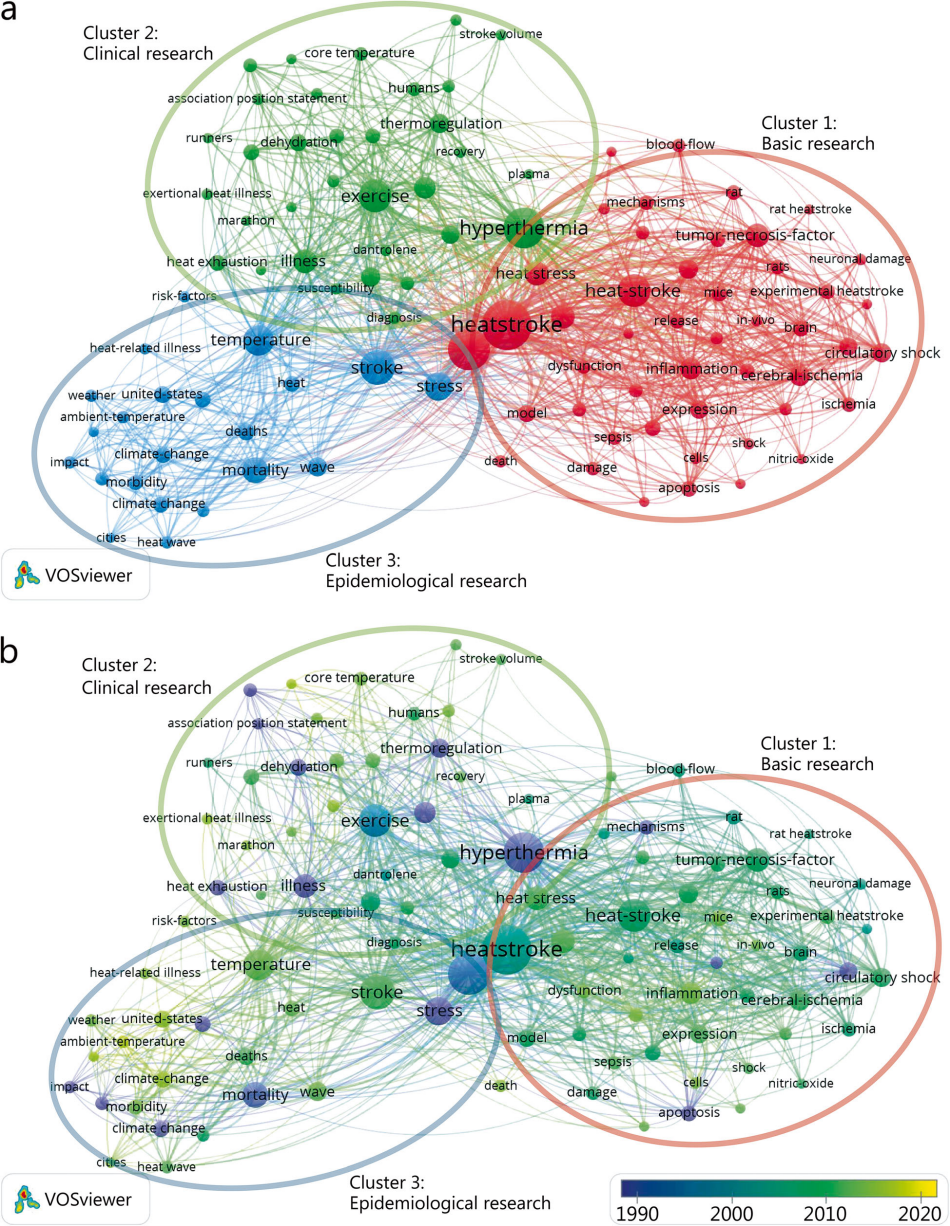
The analysis of keywords in publications on HS. **a** Mapping of the keywords in the area of HS. The words were divided into three clusters in accordance with different colors that were generated by default, specifically, basic research (left in red), clinical research (up in green), and epidemiological research (right in blue). The large icon indicates the keywords that appeared at a high frequency; **b** The distribution of keywords is presented according to the average time of appearance, with the blue representing an early appearance and the yellow indicating keywords that have appeared more recently. The smaller the distance is between two keywords, the greater the frequency of their co-occurrences

We analyzed the keywords extracted from 1443 publications using VOSviewer. As presented in Fig. 4a, 100 keywords, defined as terms that occurred more than 15 times within titles and abstracts in all papers during the analysis process, were identified and classified into three clusters, namely, basic research, clinical research and epidemiological research. Within the cluster of basic research, the following keywords were frequently mentioned: heatstroke (109 times), heat stroke (106 times), injury (94 times), heat stress (94 times), and heat-stroke (89 times). In the cluster of clinical research, relevant keywords were also listed, including hyperthermia (104 times), exercise (91 times), illness (81 times), responses (81 times), and thermoregulation (76 times). In the cluster of epidemiological research, the primary keywords were stroke (104 times), temperature (91 times), mortality (90 times), wave (64 times), and deaths (62 times). Detailed consequences with respect to the co-occurrence analysis of all incorporated keywords are presented in Supplemental Table 1.

As presented in Fig. 4b, VOSviewer colored all keywords according to the average time the word appeared. Specifically, the blue color indicates that the word appeared relatively early in the research stage, while the yellow color indicates more recent appearance. For example, during the early stage of research on HS, the AAY for hyperthermia (cluster 2), which was the major topic in this field, was 1990.1. More recently, research trends demonstrate that temperature (cluster 3), with an AAY of 2012.1, may be a new target. Within the first cluster, the newest word was inflammation (cluster 1), with an AAY of 2013.3, which occurred 65 times. In the second cluster, exercise-induced hyperthermia (cluster 2), with an AAY of 2015.4 and exertional heat illness (cluster 2) with an AAY of 2014.5, were the most recently emerging words, which appeared 17 and 21 times, respectively. For the third cluster, appearing 23 times, ambient temperature (cluster 3), with an AAY of 2016.8, rather than mortality, was noted as a new topic.

## Discussion

### Research trends of HS

It is worth noting that an analysis of HS is different from an analysis of a tumor or another disease because of the correlation between disease incidence and local climate. Heat illness, i.e., heatstroke, hyperthermia and dehydration, was responsible for 3306 of 14,539 deaths during the heat wave that occurred in France from August 1st to August 20th of 2003 [13]. Because previous studies on the association between heat exposure and HS were primarily restricted to small geographical regions, an epidemiological study of HS at the national and international levels was essential [5]. Hence, in the neighboring subtropical Sahara and Arabian deserts, the Israel Defense Forces used a heat-tolerance test (HTT) as a screening tool for the secondary prevention of exertional heat illness [14]. In addition, during the Hadj season in Mecca, Saudi-Arabia, HS threatens the health of pilgrims [15], thus specific climatic and social customs have led these countries to invest in HS research. Accordingly, data should be normalized to some extent based on related factors. Simultaneously, RRI displayed a slowly rising trend, thus demonstrating that the research demand for HS has increased year by year due to its rising incident rates.

With respect to authors, Knochel JP, who pioneered research on HS, was first author of an impressive review published in the *New England Journal of Medicine* that has been cited a number of times [2]. In addition, the cooperation among authors has been noted. For example, the map of co-authorship analysis based on WOS data reveals close cooperation among authors from English-speaking countries (Figure S1) and partly explains the mismatch between the publications and citations of Chinese researchers’ articles, a phenomenon that has called on scientists from all over the world to break through boundaries and bring about deeper cooperation. Only in this way can we simultaneously extensively affect the future development and predict the hotspots in this field.

### Research focused on HS

Published articles with the highest citation frequency are associated with correlative academic impacts in a certain field. Detailed information regarding the top ten most frequently cited publications on HS is provided in Table 2. The study published in *New England Journal of Medicine* in 2002 with Knochel JP as the corresponding author provides an exhaustive and comprehensive overview of HS and indicates that future research should focus on three areas, specifically, identifying genetic traits, searching for new biomarkers, and developing new adjuvant treatments to effectively control inflammatory reactions and counteract multiorgan complications [2]. The article published by Casa DJ presents clinical research regarding HS, while papers ranking fourth to ninth are related to epidemiological research [16]. As relatively new publications have not yet been cited much, these indicators must be interpreted with caution.

As for journals with the most publications, the focus of the top three journals was sports medicine, a result that is obviously owing to one of the two clinical types of HS, i.e., exertional heat stroke, which is associated with strenuous physical exercises (Fig. 3a).

According to the map based on the bibliographic data from the co-occurrence analysis of all keywords (Fig. 4a, b), the keywords were divided into three clusters, namely, basic research, clinical research, and epidemiological research. The density of the keywords outlined the overall structure of the map and draw attention to the most important areas of HS, revealing an even distribution among the three groups that was consistent among the three clusters [7]. However, the epidemiological research group was relatively new with respect to the publication dates. The potential reasons for this are as follows. First, the respective research in China and the United States over the past decade indicates that the research direction has transitioned towards epidemiology research (Figure S2). Furthermore, scientists from different countries have focused on the scale of heat-related diseases caused by the generation of heat waves in cities in recent years. Thus, keywords such as heat wave and cities have emerged within the epidemiological research cluster [4, 17]. However, relevant research has been limited to developed countries, such as the United States and France, suggesting that Western temperate countries must be more prepared for future heat waves [4]. Meanwhile, a well-known climatic effect known as the urban heat island, which is thought to be partly responsible for the mortality rate due to HS, has increasingly attracted the interest of researchers [18]. For example, a recent study has indicated that numerous epidemiological methods have been used to evaluate the effect of the thermal environment on mortality and morbidity and thus to estimate the mortality of temperature-attributable heat illness, which further provides new areas for epidemiological studies related to HS [19]. According to the results of the pictures, although the research direction has shifted towards epidemiological research, the corresponding input is still scarce and the relevant countries must further strengthen their research efforts (Fig. 4a, S2).

With respect to the latest research hotspots, heat-stress from the basic research cluster is the most recent (cluster 1), with an AAY of 2015.5. An increase in core temperature caused by heat stress has two possible pathways for causing multiple organ dysfunction [20]. One is that the increase in skin blood flow and the decrease in intestinal blood flow cause an elevation in intestinal epithelial barrier permeability. The immune response is then initiated, which triggers a systemic inflammatory response syndrome (SIRS). Another pathway is that the increase in core temperature causes vascular endothelial damage, followed by microvascular thrombosis and consumptive coagulation, which ultimately leads to multiple organ dysfunction. A previous study has indicated that *Escherichia coli* exerts regulation on heat shock proteins, specifically, HSP-1(heat shock protein-1), which function as stress-responsive activators [21]. Keywords such as inflammation and tumor necrosis factor (TNF) were among the most recently identified words in the related research. TNF, which is a common inflammatory factor that causes inflammation, exists in serum, tissue, and animal models [22] regardless of whether the patient has HS [23]. Hence, an elevated TNF is naturally a focus of research. As systemic inflammatory response is a precursor to multiple organ failure [20], the mechanism of systemic inflammatory response has been at the core of basic research on HS and related diseases. Furthermore, existing studies confirm that there is an overexpression of IL-1 β in the early stage of HS due to the production of inflammatory cytokines caused by the death of spleen lymphocytes under the condition of simulated HS, thus providing a new strategy for the targeted treatment of IL-1 β in the clinical treatment of HS [24, 25]. Another hotspot, permeability, is also closely related to inflammation as the gastrointestinal system plays a significant role in pathogen elimination, nutrient absorption and immunity, whereas strenuous activity weakens the intestinal integrity and impairs its barrier function [26, 27]. The intestinal hyper permeability caused by an exercise-induced increase in core temperature facilitates the bacterial translocation of endotoxin from the intestinal lumen to blood circulation, which may then result in a systemic inflammatory response and end with HS [28]. Additionally, the emergence of intestinal flora research in recent years has resulted in new perspectives [29, 30]. For example, studies have indicated that the intestinal flora and its tryptophan catabolism can enhance the intestinal barrier function [31]. Thus, investigating the changes of intestinal flora caused by HS and its effects on the intestinal barrier permeability and inflammatory response are likely to be new hotspots in future research.

Exertional heat-stroke, exercise-induced hyperthermia and exertional heat illness belong to the clinical research cluster. The emergence of the three keywords is in accordance with the existence of controversies among diagnoses, preventions and treatments of HS that arises from a reliance on core temperature for diagnosing and assessing the severity of exertional heat stroke [32]. First, a definition of HS based on a specific body core temperature value, typically > 40 °C or 40.5 °C, is not considered rigorous [1] as core temperature elevation tends to be a function of metabolic heat production during physical exercise in temperate and warm-hot conditions. However, some athletes do not show any abnormal vital signs when their body temperatures increase during such situations, thus indicating that the association between exertional heat stroke and a temperature threshold may need to be revised and improved [33]. Hence, new HS criteria that cover more sensitive organ damage markers during multiple organ failure, called Japanese Association for Acute Medicine - Heatstroke (JAAM-HS) and developed by the Japanese Association for Acute Medicine (JAAM), does not include temperature [34].

With respect to the epidemiological research cluster, the newest keywords were ambient temperature and heat waves. A study has found that the temperature significantly rises in vehicles even on clear, sunny days, regardless of whether the windows are left open, thus threatening child passenger safety [35]. HS induced by climate-related extreme heat exposure gives rise to acute mortality resulting from the exacerbations of pre-existing chronic diseases and significant increases of 0.8 °C to 0.9 °C in the global average temperature, which creates further concern for epidemics of chronic kidney disease [36]. A heat wave is characterized by a sustained period of extremely hot weather and is linked with increased mortality and morbidity, particularly among the elderly. Nonetheless, there is a lack of HS admissions during heat waves. We learn from Yan Wang et al. that understanding the spatiotemporal pattern of heat-related diseases that can attenuate the adverse impacts of heat waves is essential for protecting regional public health from the adverse effects of heat waves [5]. As a result, we recommend that necessary strategies are adopted during periods of high environmental temperatures as the induction mechanism of HS is closely related to the ambient temperature. Further research should be undertaken to investigate the combination of temperature and humidity given that heat dissipation by sweat evaporation is of importance, especially with respect to exertional heat stroke. While animal models can simulate HS by means of simulating hyperthermia body situations, the ethical issue of clinical research is doubtful, making it difficult to simulate the illness on the human body. There is abundant room for further progress in the epidemiology research of HS as its novel trends still lack sufficient attention.

## Conclusions

The United States and China were the most productive regions for research on HS, and the scientific research strength of each region was directly related to their corresponding GDP (graphic abstract). The focus of keywords gradually shifted from basic research and clinical research to epidemiological research. It was also recommended to focus on promising research hotspots, such as heat stress, tumor-necrosis factor and heat waves. Our study provides profound insights into the research history and current status of HS, which may indicate its future trend.

## Supplementary Information

**Additional file 1 **: **Supplemental Table 1.** The analytic consequence of 100 keywords with at least 16 occurrence times. **Supplemental Fig. 1.** The Co-occurrence analysis of USA and China. **Supplemental Fig. 2.** The Co-authorship analysis of organizations and countries.

## Data Availability

All data generated or analyzed during this study are included in this published article.

## Abbreviations

AAY: Average appearing year
ACA: Author co-citation analysis
CNS: Central nervous system
GDP: Gross domestic product
HS: Heat stroke
HSP-1: Heat shock protein-1
HTT: Heat-tolerance test
IF: Impact factor
IL-1 β: Interleukin-1 β
JAAM: Japanese Association for Acute Medicine
JAAM-HS: Japanese Association for Acute Medicine – Heatstroke
JCRs: Journal citation reports
RR: Relative risk
RRI: Relative research interest
SIRS: Systemic inflammatory response syndrome
WOS: Web of Science
