# Supplementary material for: Research progress of heat stroke during 1989–2019: a bibliometric analysis
Source: Mil Med Res. 2021 Jan 21;8:5. doi: 10.1186/s40779-021-00300-z (PMC7818934; doi:10.1186/s40779-021-00300-z)
Supplement: Supplementary file 1 — Additional file 1 : Supplemental Table 1. The analytic consequence of 100 keywords with at least 16 occurrence times. Supplemental Fig. 1. The Co-occurrence analysis of USA and China. Supplemental Fig. 2. The Co-authorship analysis of organizations and countries. [file 40779_2021_300_MOESM1_ESM.docx]

**Supplemental Table 1. The analytic consequence of 100 keywords with at least 16 occurrence times**

| **Keywords** | **Cluster** | **Links** | **Occurrences** | **Average appearing years (AAY)** | **Average citations** |
| --- | --- | --- | --- | --- | --- |
| heat-stress | 1 | 109 | 39 | 2015.5 | 18.20 |
| permeability | 1 | 106 | 37 | 2014.1 | 15.60 |
| cells | 1 | 40 | 19 | 2013.7 | 10.32 |
| death | 1 | 52 | 23 | 2013.7 | 12.74 |
| dysfunction | 1 | 54 | 27 | 2013.6 | 11.00 |
| activation | 1 | 48 | 27 | 2013.5 | 23.70 |
| inflammation | 1 | 69 | 68 | 2013.2 | 17.01 |
| mice | 1 | 59 | 37 | 2013.0 | 16.05 |
| in-vivo | 1 | 36 | 18 | 2012.8 | 35.72 |
| survival | 1 | 43 | 17 | 2012.1 | 18.59 |
| protein | 1 | 42 | 17 | 2011.8 | 16.47 |
| injury | 1 | 88 | 82 | 2011.3 | 19.79 |
| interleukin-6 | 1 | 43 | 16 | 2011.2 | 21.31 |
| sepsis | 1 | 49 | 27 | 2011.2 | 14.37 |
| shock | 1 | 47 | 20 | 2011.1 | 16.70 |
| hypothalamus | 1 | 50 | 25 | 2010.9 | 12.36 |
| heat stress | 1 | 88 | 82 | 2010.7 | 23.12 |
| expression | 1 | 61 | 47 | 2010.7 | 22.51 |
| experimental heatstroke | 1 | 48 | 30 | 2010.6 | 17.07 |
| cerebral-ischemia | 1 | 65 | 61 | 2009.8 | 13.89 |
| hypothermia | 1 | 59 | 28 | 2009.7 | 19.93 |
| nitric-oxide | 1 | 38 | 17 | 2009.2 | 23.06 |
| circulatory shock | 1 | 56 | 55 | 2009.0 | 16.45 |
| endotoxemia | 1 | 49 | 32 | 2008.9 | 21.50 |
| cytokines | 1 | 69 | 53 | 2008.8 | 22.17 |
| damage | 1 | 50 | 29 | 2008.7 | 17.17 |
| model | 1 | 70 | 42 | 2008.5 | 17.48 |
| whole-body hyperthermia | 1 | 54 | 30 | 2008.5 | 65.73 |
| tumor-necrosis-factor | 1 | 65 | 81 | 2008.4 | 35.05 |
| disseminated intravascular coagulation | 1 | 52 | 27 | 2008.3 | 32.04 |
| coagulation | 1 | 57 | 38 | 2007.7 | 24.13 |
| rats | 1 | 56 | 34 | 2007.6 | 23.18 |
| heat-stroke | 1 | 84 | 150 | 2007.6 | 22.01 |
| ischemia | 1 | 42 | 30 | 2007.5 | 20.13 |
| brain | 1 | 53 | 39 | 2007.2 | 22.28 |
| rat heatstroke | 1 | 36 | 16 | 2006.6 | 30.06 |
| blood-flow | 1 | 53 | 27 | 2005.6 | 36.15 |
| endotoxin | 1 | 42 | 17 | 2005.3 | 41.47 |
| fever | 1 | 45 | 20 | 2005.2 | 26.25 |
| rat | 1 | 54 | 34 | 2004.5 | 27.24 |
| release | 1 | 60 | 34 | 2004.3 | 28.29 |
| neuronal damage | 1 | 39 | 21 | 2004.2 | 35.19 |
| heatstroke | 1 | 100 | 345 | 2003.3 | 17.86 |
| cerebral ischemia | 1 | 37 | 17 | 2003.1 | 28.18 |
| heat stroke | 1 | 97 | 251 | 1993.9 | 17.63 |
| oxidative stress | 1 | 52 | 50 | 1970.6 | 22.42 |
| apoptosis | 1 | 51 | 37 | 1960.5 | 8.24 |
| exercise-induced hyperthermia | 2 | 34 | 17 | 2015.4 | 14.24 |
| exertional heat-stroke | 2 | 44 | 23 | 2014.6 | 9.39 |
| exertional heat illness | 2 | 41 | 21 | 2014.5 | 15.57 |
| marathon | 2 | 42 | 16 | 2014.3 | 16.31 |
| case series | 2 | 32 | 18 | 2013.5 | 11.89 |
| body-temperature | 2 | 47 | 22 | 2013.4 | 17.23 |
| core temperature | 2 | 42 | 28 | 2013.0 | 17.68 |
| management | 2 | 31 | 16 | 2012.8 | 19.38 |
| performance | 2 | 47 | 31 | 2012.5 | 20.16 |
| recovery | 2 | 42 | 18 | 2011.9 | 17.78 |
| exertional heat stroke | 2 | 51 | 32 | 2011.8 | 16.50 |
| prolonged exercise | 2 | 20 | 18 | 2011.4 | 57.39 |
| stroke volume | 2 | 27 | 20 | 2011.3 | 20.15 |
| susceptibility | 2 | 34 | 20 | 2011.1 | 21.60 |
| exhaustion | 2 | 37 | 16 | 2010.7 | 19.31 |
| heat illness | 2 | 52 | 39 | 2010.3 | 15.97 |
| malignant hyperthermia | 2 | 44 | 27 | 2009.7 | 23.96 |
| humans | 2 | 49 | 27 | 2009.3 | 17.81 |
| diagnosis | 2 | 35 | 24 | 2008.5 | 19.42 |
| runners | 2 | 35 | 17 | 2008.2 | 49.41 |
| exertional heatstroke | 2 | 68 | 46 | 2008.1 | 28.13 |
| rhabdomyolysis | 2 | 59 | 51 | 2007.2 | 14.61 |
| plasma | 2 | 42 | 16 | 2005.8 | 52.38 |
| dantrolene | 2 | 38 | 18 | 2002.1 | 34.67 |
| exercise | 2 | 88 | 158 | 1996.8 | 17.49 |
| hyperthermia | 2 | 98 | 234 | 1990.9 | 20.19 |
| illness | 2 | 79 | 79 | 1987.8 | 17.96 |
| responses | 2 | 77 | 69 | 1980.7 | 25.43 |
| thermoregulation | 2 | 73 | 53 | 1972.5 | 17.89 |
| dehydration | 2 | 50 | 40 | 1958.5 | 31.75 |
| heat exhaustion | 2 | 52 | 37 | 1951.3 | 38.38 |
| cold-water immersion | 2 | 36 | 27 | 1940.6 | 16.59 |
| association position statement | 2 | 34 | 21 | 1919.3 | 14.48 |
| ambient-temperature | 3 | 31 | 23 | 2016.8 | 14.91 |
| heat waves | 3 | 30 | 17 | 2015.5 | 14.35 |
| climate-change | 3 | 42 | 35 | 2015.1 | 33.29 |
| united-states | 3 | 49 | 45 | 2014.8 | 27.73 |
| heat-related illness | 3 | 40 | 19 | 2013.5 | 13.84 |
| cities | 3 | 25 | 17 | 2013.5 | 25.53 |
| risk-factors | 3 | 38 | 21 | 2013.3 | 28.95 |
| weather | 3 | 40 | 25 | 2013.0 | 52.60 |
| morbidity | 3 | 34 | 30 | 2012.8 | 62.57 |
| temperature | 3 | 83 | 136 | 2012.1 | 27.51 |
| wave | 3 | 62 | 52 | 2012.1 | 22.54 |
| heat | 3 | 57 | 37 | 2012.0 | 30.97 |
| heat wave | 3 | 31 | 23 | 2011.1 | 63.43 |
| stroke | 3 | 97 | 170 | 2010.5 | 19.35 |
| deaths | 3 | 63 | 42 | 2010.4 | 51.81 |
| chicago | 3 | 35 | 24 | 2008.3 | 56.00 |
| mortality | 3 | 82 | 100 | 1991.9 | 35.57 |
| stress | 3 | 90 | 111 | 1974.8 | 25.05 |
| climate change | 3 | 35 | 36 | 1958.4 | 27.83 |
| risk | 3 | 50 | 35 | 1898.0 | 23.46 |

**Supplemental Fig 1. The Co-occurrence analysis of USA and China.**

**
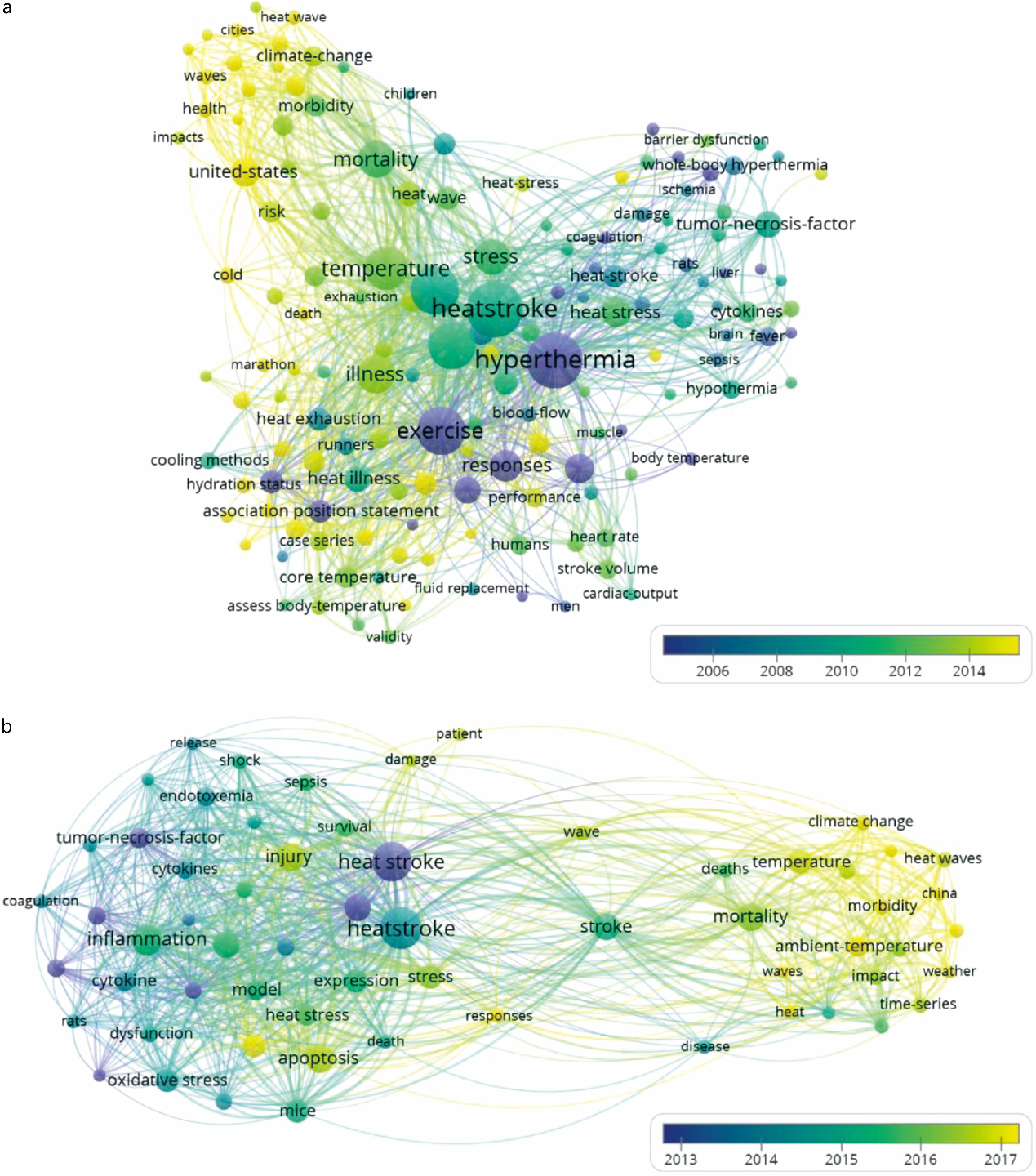
**

**Supplemental Fig 2. The Co-authorship analysis of organizations and countries.**

**
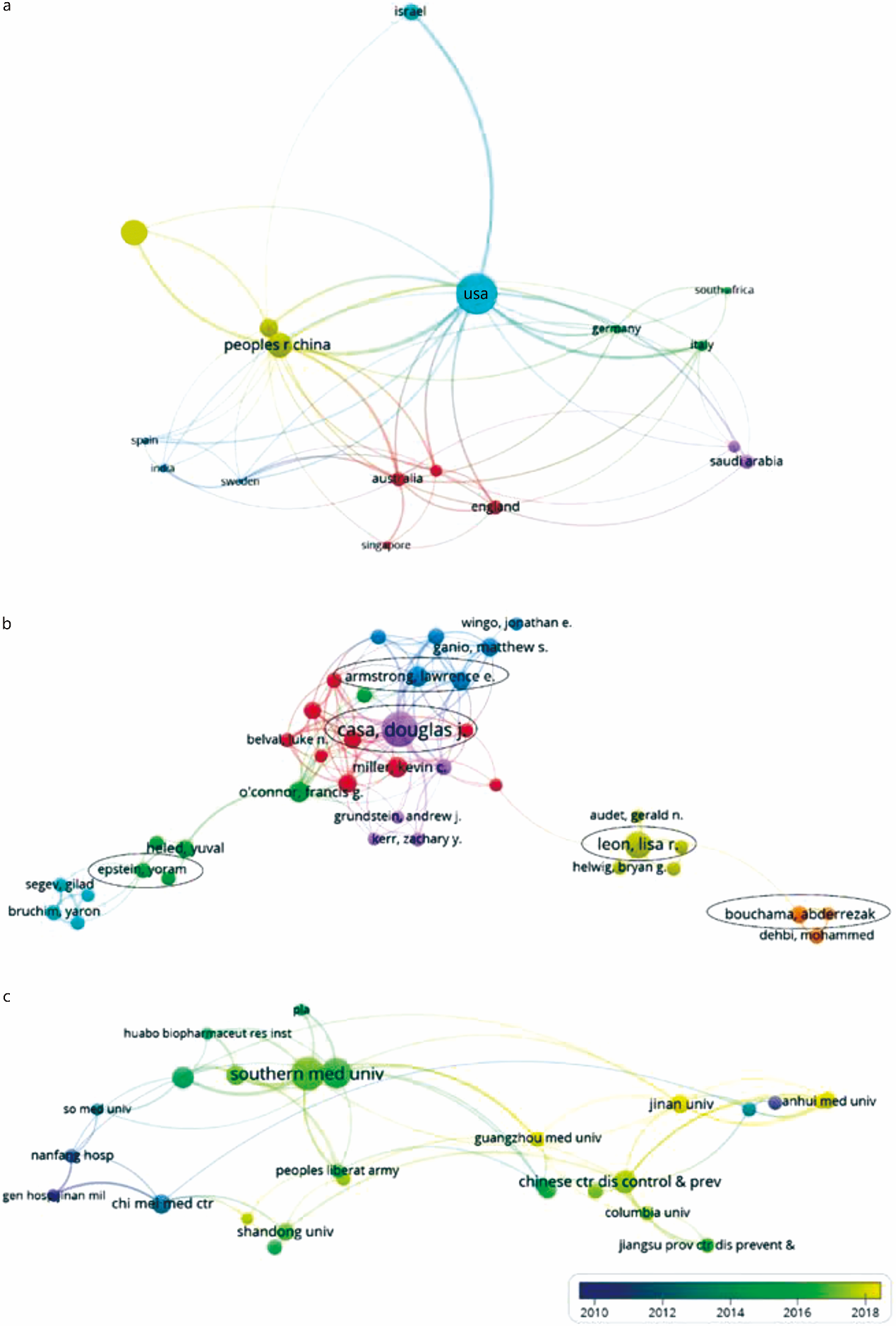
**
